# A multi-level explanatory-sequential mixed-methods study of perinatal toxicology practices in New York State: Protocol

**DOI:** 10.1371/journal.pone.0339284

**Published:** 2025-12-31

**Authors:** Sugy Choi, Elizabeth Knopf, Erin Kim, Charles J. Neighbors, Carolyn A. Berry, Erinn Hade, Chau Trinh-Shevrin, Mishka Terplan, Neil S. Seligman, David J. Garry, Jennifer McNeely

**Affiliations:** 1 Department of Population Health, New York University Grossman School of Medicine, New York, New York, United States of America; 2 Friends Research Institute, Baltimore, Maryland, United States of America; 3 University of Rochester Medicine, Rochester, New York, United States of America; 4 Stony Brook Medicine University School of Medicine, Stony Brook, New York, United States of America; PLOS: Public Library of Science, UNITED KINGDOM OF GREAT BRITAIN AND NORTHERN IRELAND

## Abstract

**Objective:**

Maternal morbidity and mortality (MMM) rates from drug overdoses have increased, especially among pregnant and postpartum women aged 35–44. However, there is limited understanding of how current toxicology testing practices are implemented in hospital settings and how well they support, or undermine, linkage to care. The goal of the study is to understand variations in toxicology testing use among pregnant and postpartum women, explore hospital- and individual-level differences, and assess outcomes.

**Methods:**

Using the Socio-cultural Framework for the Study of Health Service Disparities (SCF-HSD) we will perform a mixed-methods study to understand testing policies and practices in NY State.

Aim 1 will employ multilevel statistical models using New York State Medicaid claims data (2021–2024) to identify predictors of perinatal toxicology testing and characterize hospital-level variation across hospitals. Aim 2 will involve one-on-one interviews with hospital administrators and clinical staff to document and analyze testing policies and practices, capturing diverse perspectives on testing rationales, attitudes, and adherence. Aim 3 will integrate quantitative and qualitative evidence through a mixed-methods design, incorporating perspectives of individuals with lived experience, via focus group sessions to inform and refine hospital policy recommendations.

**Discussion:**

Our findings will inform how to improve disparities in toxicology testing for pregnant and postpartum women. Addressing these challenges requires shifting emphasis toward standardized, evidence-based toxicology testing protocols, strengthening pathways to supportive services, and advancing policy reforms that reduce stigma and inequities in care.

## Introduction

Maternal morbidity and mortality (MMM) from drug overdoses have increased. From 2018 to 2021, overdose mortality more than tripled among pregnant and postpartum women ages 35–44 [1]. Yet, effective approaches to substance use disorder (SUD) identification and intervention for this population remain underdeveloped. The National Institutes of Health (NIH) acknowledges the importance of reducing preventable maternal deaths, lessening the impact of severe maternal complications, and advancing health equity across communities [2]. The perinatal period provides a critical opportunity to address the needs of women who have SUD and to provide appropriate healthcare and related treatment services [3–7]. However, stigma and other barriers, such as legal implications of a positive toxicology (i.e., testing for drug use) result, hinder access to healthcare services for perinatal women with SUD, leading to reduced use or avoidance of perinatal care and SUD treatment services [5,8–17].

Although validated self-reported screening tools are recommended for assessing substance use during pregnancy, they are inconsistently administered across clinical settings. In contrast, many providers continue to rely on toxicology testing, despite its limitations, including lack of standardization, potential for false positives, and ethical concerns around consent and punitive consequences. Prior research highlights that toxicology testing policies vary widely across institutions, raising concerns about inconsistent clinical decision-making and the potential for unequal treatment of pregnant patients [18–20]. Such variation and potential overuse of testing may undermine the effectiveness of recommended screening practices [5,6], shifting the focus from initiating SUD treatment to reporting, and in turn creating barriers to prenatal care when applied variably or perceived as punitive. [21,22]. At the same time, hospitals are tasked with balancing clinical, legal, and ethical considerations around testing, often without clear evidence to guide practice [23,24]. The resulting landscape is one in which maternal health outcomes may be undermined by fragmented practices for identifying substance use that can exacerbate mistrust of the healthcare system among women who use opioids and other drugs.

Prior research has documented disparities in toxicology practices among pregnant women, revealing an absence of clear and standardized guidelines [19,22,24–31]. Studies indicate disparities in reporting and uncertain predictive utility [19,23,26,32–39], primarily focusing on patient-level factors, mostly among Black and White women, without addressing the complex multilevel context, such as institutional policies, provider discretion, regional legal frameworks, and structural racism, that shape how and when toxicology testing is administered. Investigations into health disparities have often been limited to specific institutions or health systems, with a scarcity of high-quality race and ethnicity data for affected populations [26,40–42]. Recent literature emphasizes the need for mixed-methods, such as focus groups with hospital administrators and interviews with patients, to inform best practices and develop guidelines for toxicology practices [18].

We are conducting a mixed-methods study to examine multi-level variation in the use of toxicology testing among pregnant and postpartum women, with the goal of understanding how these practices differ across institutions and how they relate to patient outcomes. This paper presents the study protocol. This research builds on prior studies by identifying multilevel predictors using administrative data, analyzing hospital policies, and gathering insights from providers through one-on-one interviews and focus group sessions in New York State (NYS).

### Objectives

The study will proceed in three phases: (1) identify multilevel factors contributing to disparities in perinatal toxicology testing and characterize hospitals by testing patterns and demographic profiles across NYS from 2021–2024; (2) conduct one-on-one interviews with hospital administrators and staff to examine testing policies, practices, and perspectives on adherence; and (3) integrate quantitative and qualitative findings using a mixed-methods approach, incorporating the perspectives of individuals with lived experience through focus group interviews.

## Materials and methods

### Study design and setting

This study uses an explanatory-sequential mixed-methods design with three complementary components: (Aim 1) retrospective analysis of NYS Medicaid data, (Aim 2) semi-structured interviews with hospital staff, and (Aim 3) focus groups with individuals with lived experience. Quantitative and qualitative data will be integrated to synthesize findings examining how hospital practices and policies impact perinatal toxicology testing and contribute to health disparities. The study will take place in NYS, incorporating both urban and rural hospitals and birthing centers to capture a wide range of institutional contexts and community experiences. This study will be conducted at New York University (NYU) Grossman School of Medicine. The protocol of this study was approved by the NYU Langone Health Institutional Review Board (i25-00835). The reporting of this study will comply with the standard protocol items recommended for observational studies (STROBE) [43] and qualitative studies (COREQ) [44].

#### Conceptual framework.

**Fig 1:**
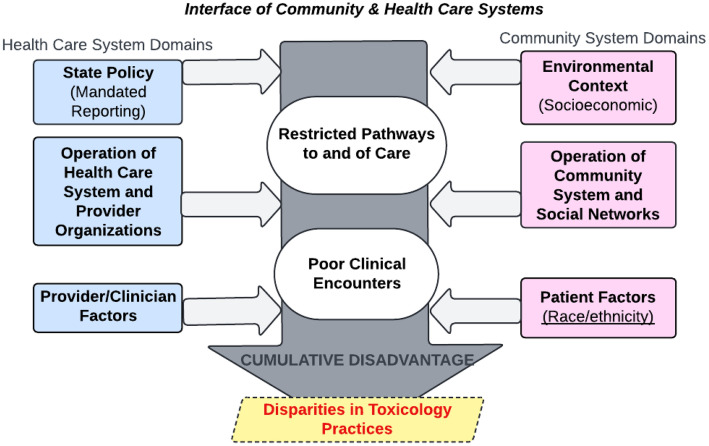
Adapting SCF-HSD to examine disparities in toxicology practices.

The study is guided by the Socio-cultural Framework for the Study of Health Service Disparities (SCF-HSD) [45], adapted to examine disparities in toxicology testing practices among perinatal patients from both healthcare system and community perspectives (Fig 1). This model emphasizes how factors at multiple levels, individual patient characteristics, provider/clinician behavior, organizational policies, and broader societal contexts such as policy and community resources, interact to influence differences in testing rates. For example, individual patient factors, including Black racial identity, have been associated with higher rates of toxicology testing, while organizational policies and systemic factors, such as healthcare access and socioeconomic disparities, also affect service quality. The framework highlights how cumulative disadvantages across these domains, such as suboptimal clinical encounters influenced by multilevel factors, can disproportionately impact minority populations. This study’s mixed-methods approach, which integrates quantitative analyses of multilevel predictors and hospital variation with qualitative insights into personal experiences and contextual factors, provides a rich understanding of how structural and social determinants contribute to disparities in perinatal toxicology testing.

### Aim 1 study data and sample

For Aim 1, the study population will be derived from NYS Medicaid claims data through the Medicaid Data Warehouse and includes all reproductive aged females (18−45) who have had at least one pregnancy-related claim in the last 12 months from 2021 to 2024 and who are enrolled in the NYS Medicaid program. We will exclude patients with insurance enrollment gaps of more than 45 days in the prior 12 months during pre-pregnancy to ensure continuity of coverage. To link pregnant patients to their infants, we will use case numbers assigned to individuals who apply for Medicaid together. Additionally, the team will match demographic information such as last name and address, as well as specific delivery hospitalizations identifying delivery dates (ICD-10: Z37.0, Z37.2, Z37.3, Z37.5, Z37.6) that occur within 30 days + /- the child’s date of birth [46,47]. This matching process enables us to identify mother-infant dyads with a high level of accuracy (The team achieved a match rate of 94% for infants born between 2016 and 2021). From this base study population, we will identify a subpopulation of patients with SUD involving alcohol, opioids, cocaine, amphetamines, cannabis, and other drugs. The SUD subpopulation will be identified using ICD-10 diagnosis codes, where patients with two or more primary care or outpatient diagnoses or one inpatient diagnosis in the study pre-period (12 months prior to pregnancy) will be classified as having that condition. After applying all exclusion criteria, the final study population will include women and their infant(s) residing in NYS from the base study cohort.

### Aim 1 study measures

Pregnant women will be classified into the following race/ethnicity categories: non-Latinx White, Black, American Indian/Alaska Native, Asian, and Latinx. It is important to note that race/ethnicity categorizations are socially constructed, and health disparities among different racial/ethnic groups are influenced by systemic oppression and marginalization, with race/ethnicity serving as a proxy [48,49]. Classification of these patients based on their race/ethnicity will occur so that we can explore whether toxicology practices during the perinatal period varies by race/ethnicity and if the association between ZIP Code and toxicology practices changes when race/ethnicity are considered. Thus, we will also use ZIP Codes to confirm and validate findings regarding health disparities. Other patient-level predictors will include demographic characteristics, such as indicators of socioeconomic status derived from Medicaid data (e.g., eligibility category, enrollment in TANF or SNAP). Clinical factors will also be included, such as maternal and infant health conditions identified through Medicaid claims. These will be captured using ICD-10 diagnosis codes, procedure codes, and pharmacy claims to identify chronic conditions (e.g., hypertension, diabetes), behavioral health diagnoses (e.g., depression, SUD), and pregnancy-related complications. In addition to individual-level Medicaid data, we will incorporate ZIP Code-level information to contextualize findings geographically. This will allow us to link records to American Community Survey (ACS) data, providing neighborhood-level indicators of socioeconomic status, such as median household income, educational attainment, unemployment rates, housing stability, and access to transportation [50]. The use of CDC Social Vulnerability Index [49], which is a composite index of multiple Census community-level measures, will be explored. Hospital-level characteristics, such as size (e.g., number of maternity beds), type (teaching vs. community), and urban/rural location, will be drawn from the NYS Department of Health Facility Data.

### Aim 2 study design and participants

Aim 2 will use qualitative methods to explore hospital-level decision-making and practices that may contribute to disparities in perinatal toxicology testing. Within each selected hospital, we will conduct semi-structured interviews with key stakeholders who have experience working with pregnant women, including obstetric providers, nurses, social workers, and hospital administrators, to understand institutional policies, workflows, and perceptions influencing toxicology testing.

### Aim 2 study procedures

#### Recruitment.

Based on the hospital-level deviation metric developed in Aim 1, we will purposively sample hospitals that exhibit the largest positive or negative deviations from expected testing patterns.

#### Data collection.

Interview guides will be informed by prior literature on structural barriers, clinical decision-making, and patient-provider interactions. The NYULH study team will obtain verbal informed consent from all participants prior to semi-structured interviews. Participants will receive an overview of the study purpose, procedures, potential risks, and benefits. Interviews (60–90 minutes) will be conducted virtually via Zoom or in person, with audio and video recording; video files will be immediately deleted, and audio files will be securely transcribed. Transcripts will use study IDs only, without direct identifiers. A brief demographic questionnaire will be completed at the end of each session. Findings from Aim 2 will provide insight into contextual factors driving non-clinical variation in testing, complementing the quantitative patterns identified in Aim 1. All participants will receive detailed information about the study and provide informed consent prior to participation. Participation will be voluntary, and all participants will be compensated for their time and contributions ($50).

### Aim 3 study design and participants

Aim 3 uses a qualitative approach to gather insights from individuals with lived experience of perinatal toxicology testing. Participants will be recruited through community organizations, clinics, and social media outreach, with eligibility limited to adults (18+) who experienced toxicology testing during pregnancy or postpartum. Two to three focus groups, each with 4–6 participants, will be conducted in person or virtually, lasting approximately 60–90 minutes. A trained facilitator will use a semi-structured guide to explore experiences with testing, perceived drivers (clinical vs. non-clinical), and its impact on care and trust. Preliminary findings from Aims 1 and 2 will be shared during the sessions to solicit participant feedback and contextualize quantitative patterns.

### Aim 3 study procedures

#### Recruitment.

Recruitment will be conducted using purposive sampling in partnership with community-based organizations, harm reduction programs, and clinical partners across NYS. These partners bring deep knowledge of their communities and are well-positioned to identify settings and populations where recruitment is likely to be most successful [51]. Recruitment materials and procedures will be designed to be culturally responsive and trauma-informed, emphasizing confidentiality, respect, and the voluntary nature of participation. A combination of flyers, provider referrals, peer outreach, and word-of-mouth recruitment strategies will be used. Interested participants will be screened for eligibility based on inclusion criteria (e.g., reproductive age, recent pregnancy, and recent substance use experience).

#### Data collection.

Those eligible participants will be provided detailed study information and will give informed consent prior to enrollment. The NYULH study team will obtain verbal informed consent from all participants prior to semi-structured focus groups. Participants will receive an overview of the study purpose, procedures, potential risks, and benefits. Focus groups will be conducted virtually via Zoom or in person, with audio and video recording; video files will be immediately deleted, and audio files will be securely transcribed. For study materials, we will develop visual aids such as slides and handouts summarizing findings, along with structured feedback forms to facilitate organized input. Focus groups will last approximately 90–120 minutes each, and participants will be compensated for their time and contributions ($50).

### Data analysis

For Aim 1, data analysis will focus on identifying multilevel predictors of disparities in perinatal toxicology testing using NYS Medicaid data. We will begin by using hierarchical generalized linear models to examine predictors of testing during the perinatal period, accounting for individual-, clinical-, and hospital-level characteristics such as maternal race/ethnicity, age, Medicaid eligibility, clinical risk factors, and geographic region. Next, we will assess hospital-level variation by calculating risk-adjusted average testing rates for each hospital, stratified by race and ethnicity and other demographic variables. To further understand institutional-level differences, we will develop a hospital-level deviation metric that quantifies the extent to which observed testing practices differ from expected patterns based on clinical need. Clinical need for toxicology testing was operationalized as the presence of a documented SUD or clinical indicators suggestive of substance use (enrollment in medication for opioid use disorder programs, clinical documentation of substance use or behavioral indicators, e.g., overdose, withdrawal symptoms). First, we will calculate the overall rate of toxicology testing across all hospitals to establish a global baseline. This rate can serve as a reference point for evaluating institutional variation. We will conduct a multivariate logistic regression model to estimate the expected probability of toxicology testing for each patient. The model output will present as the predicted probability of testing based on clinical and structural factors. For each hospital, we plan to calculate: Deviation = Observed rate (proportion of patients tested) – Expected rate (mean predicted probability of testing). This metric will allow us to identify hospitals and regions where non-clinical factors may influence testing decisions. To explore potential disparities, we will stratify the data by demographic variables including race, sex, and age group. Within each subgroup, we will compare observed and expected testing rates and calculated deviation metrics to identify patterns suggestive of hospital/provider behaviors. Findings from this aim will inform the qualitative follow-up in Aim 2 by highlighting institutions with substantial variation and will contribute to the development of strategies to reduce variations in perinatal toxicology practices.

For Aims 2 and 3, to ensure accurate documentation of participant feedback, audio recordings and written notes will be taken. If identifying information is shared during the interviews and focus group sessions, all identifiable information will be redacted in the written transcript. The identifiers will be kept confidential by being kept separate from the recordings and interview notes. Instead, the recordings and transcripts will be labeled with a unique study code. The research team will not be able to track transcripts back to an individual’s name and affiliation after the transcription process and destruction of identifiers.

The research team will use Dedoose, a web-based application for qualitative and mixed-methods data analysis (Aims 2 and 3), to analyze the interview and focus group data. Dedoose will help the research team efficiently categorize and analyze the responses, ensuring a faster and systematic approach to the data. All transcripts will be analyzed using thematic analysis with both deductive and inductive coding. We will employ strategies to ensure trustworthiness and rigor, including double-coding, member checking, and maintaining an audit trail.

#### Integration of quantitative and qualitative data.

**Fig 2:**
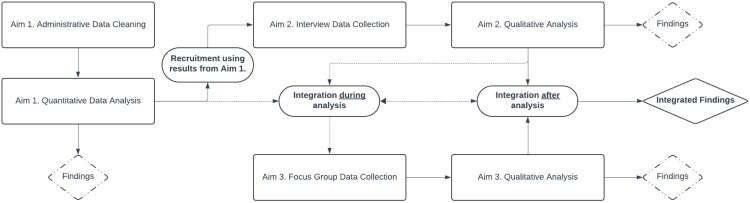
Integration of quantitative and qualitative data.

Aim 3 will integrate findings from Aims 1 and 2 to identify actionable strategies to reduce disparities in perinatal toxicology testing by conducting focus group sessions with current or former patients with lived experience (Fig 2). This approach fosters a supportive environment where participants can share their experiences, perspectives, and concerns in dialogue with others who have faced similar circumstances. The group dynamic encourages reflection, validation, and the emergence of shared themes that may not surface in individual interviews [52]. Using a mixed-methods explanatory sequential approach and SCF-HSD framework, we will synthesize quantitative results on multilevel predictors and hospital variation (from Aim 1) with qualitative insights from stakeholder interviews (from Aim 2) to inform focus group guides and materials. Themes and insights derived from Aim 2 about hospital policies, provider attitudes toward toxicology testing, and implementation challenges will inform the focus group discussion guide in Aim 3, thereby ensuring relevance to real-world practices. The iterative feedback process will involve ongoing engagement with key stakeholders, including patients, clinicians, and hospital administrators*,* at multiple stages of the project. This will ensure that practice/policy recommendations are grounded in both empirical evidence and real-world experiences, enhancing their relevance, acceptability, and feasibility for addressing variations in perinatal toxicology testing and care. Sharing preliminary results and language with perinatal individuals will help illuminate what practices/policies are perceived as helpful or harmful for treatment engagement and care navigation.

### Status and study timeline

This study will start August 2025 and will end July 2030. First, in year 1, we will obtain and analyze Medicaid data to identify multilevel factors contributing to disparities in toxicology practices during the perinatal period. We will begin characterizing hospitals by testing patterns as well as race and ethnicity profile across NYS from 2021 to 2024 (Aim 1). At the time of manuscript preparation, we are in the process of executing the DUA governing access to the retrospective dataset. As a result, the specific date of data access and the extent of author access to identifiable participant information remain undetermined. Next, in Year 2, we will continue (Aim 1) analysis, recruit and interview hospital staff, and collect and analyze toxicology testing policies and practices (Aim 2). Then, Year 3, we will integrate quantitative and qualitative findings from (Aim 1) and (Aim 2) using a mixed-methods design, incorporating the perspectives of perinatal women who use substances via focus group sessions to develop hospital-level policy recommendations (Aim 3). In Year 4, the team will analyze the focus group data, develop policy recommendations, and submit manuscripts. Lastly, in Year 5, we will disseminate study findings as well as prepare and submit an R01 grant application.

### Ethics approval

Ethics approval was obtained from the NYU Langone Health IRB (#i25-00835). This study involves minimal risk to participants. Aim 1 involves the analysis of retrospective Medicaid data, which presents no or minimal risk to participants. The research team will access the data under established data use agreements and will follow strict protocols for secure storage and handling. For Aims 2 and 3, potential risks are minimal and primarily involve mild emotional discomfort when discussing sensitive topics related to perinatal toxicology practices and disparities. The research team will create a supportive, non-judgmental environment, ensuring participants can skip questions, take breaks, or withdraw at any time without consequence. All interviews and focus group data will be coded and securely stored on password-protected systems, and results will be reported in aggregate to protect confidentiality. Participants will have access to support resources if needed, and summaries of key insights and policy recommendations will be shared for feedback. Given that the study involves perinatal women, a potentially vulnerable population, the research team will prioritize respect, beneficence, and justice throughout the study. Informed consent will clearly outline study procedures, risks, benefits, and participant rights, and measures will be taken to safeguard autonomy, privacy, and well-being at all stages. Participants in virtual 1:1 interviews and focus groups will provide verbal consent; written consent will be collected for in-person activities. For remote or telephone interactions, verbal consent will be documented in a secure tracking system. Use of de-identified administrative data will not require individual consent and will comply with institutional and state DUA. Retrospective data will be accessed after a signed DUA. DUA with the Department of Health will safeguard privacy and confidentiality. Although risks are minimal, research staff are trained to monitor distress and offer participants the option to pause, skip questions, or withdraw. Those in distress will be offered immediate support or referral.

## Discussion

This study is designed to provide a comprehensive understanding of disparities in perinatal toxicology testing by integrating quantitative analyses of statewide Medicaid data with qualitative insights from hospital staff and perinatal women. By examining multilevel predictors of testing, including patient, provider, organizational, and societal factors, the study addresses gaps in knowledge regarding the structural and systemic determinants of disparities in care. The development of a hospital-level deviation metric will allow for the identification of institutions and regions where non-clinical factors may influence testing practices, providing actionable targets for quality improvement and policy interventions.

Findings from this study have several practical implications. They can inform the development of evidence-based hospital policies and clinical guidelines aimed at reducing variation in toxicology testing and supporting patient-centered care. Results may also guide provider training to improve provider practices and inform health system interventions that reduce structural barriers to care. By engaging perinatal women in the research process, policy recommendations will be grounded in patient experiences and tailored to meet the needs of impacted communities.

Future research could build on these findings by testing targeted interventions in hospitals with high deviation scores or by exploring longitudinal outcomes of perinatal individuals impacted by differential testing practices. Additionally, methods developed in this study, such as the hospital-level deviation metric, may be adapted to examine disparities in other clinical practices or populations. Overall, this study has the potential to advance understanding of health service disparities, inform actionable strategies to promote evidence-based policies, and contribute to improved maternal and infant outcomes across diverse populations.

Overall, this mixed-methods approach will allow us to contextualize statistical patterns of toxicology testing within the lived experience and institutional realities of care delivery. We will identify specific hospital practices, structural barriers, and modifiable policies that contribute to differences in testing and prioritize them based on feasibility and potential impact. Following completion of this study, the integrated findings may be used to develop evidence-based recommendations for clinical practice guidelines, provider training, and policy interventions aimed at reducing disparities in perinatal toxicology testing across NYS. Additionally, we will share findings with participating hospitals, state health agencies, and community stakeholders to facilitate implementation and dissemination of best practices.

### Strengths and limitations

This study leverages a mixed-methods design to provide a comprehensive understanding of variations in perinatal toxicology testing practices. The integration of quantitative analyses of Medicaid data with qualitative insights from hospital staff and perinatal women allows for examination of both multilevel predictors and contextual factors influencing testing practices and its downstream effects. The use of statewide administrative data ensures a large, diverse sample, enhancing generalizability across NYS. Additionally, the study applies a robust conceptual framework (SCF-HSD) to guide interpretation and policy relevance, emphasizing variations and cumulative disadvantage. The inclusion of perspectives from both healthcare providers and perinatal individuals strengthens the relevance and applicability of findings to real-world practice and policy.

Potential limitations include reliance on administrative data, which may lack detailed clinical nuance and may be subject to coding errors or incomplete information [51]. Race and ethnicity data in Medicaid administrative datasets are often incomplete, inconsistent, and may not accurately capture individuals with multiple racial or ethnic identities [53]. Additionally, the observational design of the quantitative analyses precludes causal inference, and residual confounding by unmeasured factors may remain. Qualitative data may be influenced by self-report bias or social desirability, particularly when discussing sensitive topics such as discrimination or institutional practices. The sample size for interviews and focus groups is limited, which may constrain the transferability of findings to all hospitals or perinatal populations. The generalizability of these findings may be limited to other regions, as the study is based on one state. However, because the mixed-methods analysis is on how hospital practices, decision-making processes, and attitudes influence testing, we anticipate that findings may translate to hospitals outside of NYS. This approach will allow us to interpret the results appropriately, considering both the statistical trends and the nuanced perspectives of the patients involved [54–56]. Despite these limitations, the combined quantitative and qualitative approach provides a rich and rigorous assessment of variation in perinatal toxicology practices in NYS hospitals.

### Dissemination plans

Final study results and recommendations will be shared with stakeholders, including hospital administrators, the research community, and policymakers, to guide future policy changes and research. Peer-reviewed manuscripts will be submitted to journals in the fields of public health, maternal and child health, addiction, and health disparities. Findings will also be presented at regional and national conferences (e.g., Addiction Health Services Research, AcademyHealth, and others).

In addition, based on the integrated findings, we will develop actionable policy recommendations that address identified variations and systemic factors influencing toxicology testing practices. We hope these recommendations will propose changes to hospital policies for standardized implementation of testing across diverse populations, alongside training programs for staff to mitigate practice differences.

### Amendments to the study

This study may be suspended or prematurely terminated if there is sufficient reasonable cause. Written notification, documenting the reason for study suspension or termination, will be provided by the suspending or terminating party to Principal Investigator (PI) Dr. Sugy Choi, NIH, and other regulatory authorities. If the study is prematurely terminated or suspended, the PI will promptly inform the IRB and will provide the reason(s) for the termination or suspension.

Circumstances that may warrant termination or suspension include, but are not limited to, determination of unexpected, significant, or unacceptable risk to participants, insufficient compliance with protocol requirements, and determination of futility. The study may resume once concerns about safety, protocol compliance, and data quality are addressed and satisfy the sponsor and/or IRB.
